# Isolated Acute Dysphagia as a Probable Rare Presentation of Guillain–Barré Syndrome with Complete Recovery: A Case Report

**DOI:** 10.3390/geriatrics9040090

**Published:** 2024-06-28

**Authors:** Soo Ho Lee, Ji Yoon Jung, Mi-Jeong Yoon, Joon-Sung Kim, Bo Young Hong, Sun Im, Yeun Jie Yoo

**Affiliations:** 1 Department of Rehabilitation Medicine, St. Vincent’s Hospital, College of Medicine, The Catholic University of Korea, Suwon 16247, Republic of Korea; hossu0801@gmail.com (S.H.L.); jennylidada@gmail.com (J.Y.J.); allogen@naver.com (M.-J.Y.); joonsungg.kim@gmail.com (J.-S.K.); byhong@songeui.ac.kr (B.Y.H.); 2 Department of Rehabilitation Medicine, Bucheon St. Mary’s Hospital, College of Medicine, The Catholic University of Korea, Bucheon 14647, Republic of Korea; lafolia@catholic.ac.kr

**Keywords:** Guillain–Barré Syndrome, acute bulbar palsy, dysphagia, video fluoroscopic swallowing study, immunotherapy

## Abstract

Dysphagia is prevalent among the elderly and can lead to serious complications, often manifesting as a clinical symptom of various neurological or muscular pathologies, including Guillain–Barré Syndrome (GBS). GBS is an acute immune-mediated polyradiculoneuropathy, and dysphagia may arise during its course due to cranial nerve involvement. In rare GBS variants, dysphagia may present as the initial or sole clinical manifestation, posing diagnostic challenges. In this study, we present the case of an elderly female patient with dysphagia, eventually diagnosed with an atypical variant of GBS. Initially, the patient required nasogastric tube feeding; however, complete recovery was achieved through immunotherapy. This case underscores the importance of clinicians conducting thorough evaluations of factors influencing the swallowing mechanism and remaining vigilant about identifying uncommon causative factors. Such approaches enable the implementation of effective disease-modifying therapies, potentially leading to the resolution of dysphagic symptoms.

## 1. Introduction

Dysphagia is highly prevalent among the elderly, affecting approximately 30.5% of seniors living in the community and 53.6% to 58.6% of those residing in nursing homes [1]. Dysphagia can lead to serious complications in the elderly, including aspiration pneumonia, asphyxia, and malnutrition. Swallowing involves a complex process necessitating the coordination of central, peripheral, and autonomic nervous systems to effectively regulate oropharyngeal muscles [2]. Consequently, dysphagia can serve as a clinical manifestation of various neurological or muscular pathologies, including stroke, inflammatory myositis, myasthenia gravis, botulism, and Guillain–Barré Syndrome (GBS) [3]. Treatment approaches and prognosis for dysphagia depend on the causative disease. While autoimmune neurogenic dysphagia is uncommon, the potential for improvement through immunotherapy underscores the importance of not overlooking this condition [4].

GBS is an acute immune-mediated polyradiculoneuropathy with a classic presentation of rapidly progressive muscle weakness that typically starts in the lower limbs and can ascend to involve the upper limbs, and dysphagia may occur during the course of the disease due to cranial nerve involvement [5]. Diagnosis is not typically challenging, but atypical variants often result in incorrect diagnoses or delayed treatments. For example, in the case of rare variants such as the pharyngeal-cervical-brachial (PCB) variant or acute bulbar palsy (ABP)-plus syndrome, prominent bulbar palsy may be the first or sole clinical manifestation [5,6]. Patient-specific factors, such as age >60 years, pre-existing medical diseases, and atypical clinical presentations, have been recognized as factors significantly associated with both delayed diagnosis and increased mortality in cases of GBS [7].

In this study, we report a case of an elderly female patient that suffered from isolated acute-onset dysphagia, who was diagnosed with an atypical variant of GBS and completely recovered with immunotherapy.

## 2. Case Description

The patient was informed about the Informed Consent Statement and provided their consent for the publication of this case article and any accompanying images.

A 76-year-old woman presented to our hospital’s emergency room with sudden-onset dysphagia during meals. She experienced difficulty swallowing, frequent aspirations, and regurgitation of food into her nose, particularly when ingesting large amounts. It was her second episode of dysphagia. Three years ago, she experienced transient dysphagia, which spontaneously resolved after a week. She had a history of left posterior cerebral artery infarction 9 years ago, but there were no neurological sequelae. She denied any recent history of vaccination, infective illness such as upper respiratory tract infection, or diarrhea prior to the onset of symptoms. She complained of a hoarse voice; nonetheless, a laryngoscopic examination revealed no specific abnormalities. Apart from the bilateral loss of the gag reflex, other cranial nerve functions appeared intact. There was no weakness in the neck or limb muscles, and the sensory examination was normal. Deep tendon reflexes were normal in the upper limbs and mildly decreased in the lower limbs, without signs of pathologic reflexes. Initial laboratory findings showed no evidence of inflammation, and no ECG abnormalities were found. Brain MRI with contrast enhancement revealed no new lesions associated with her symptoms, and she was subsequently referred to our outpatient clinic for further evaluation and treatment of dysphagia.

**Figure 1:**
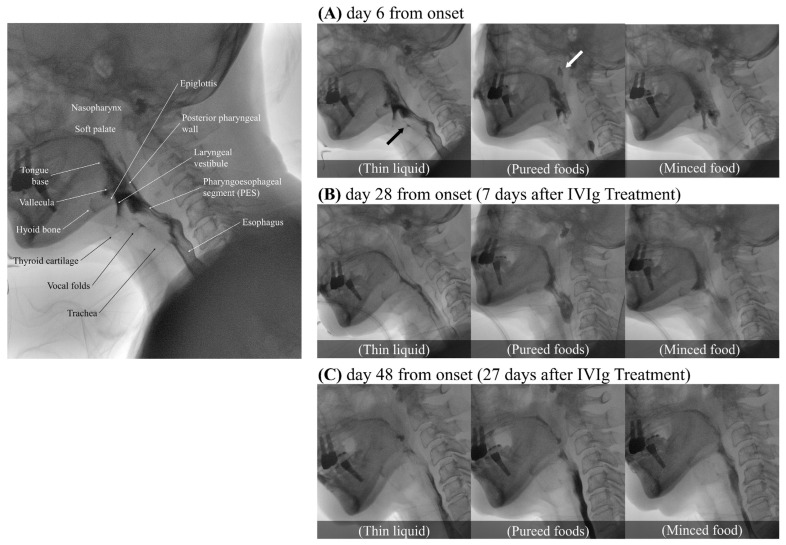
Serial Video Fluoroscopic Swallowing Study (VFSS) images of the patient on days 6, 28, and 48 from the onset of the illness, utilizing thin liquid (International Dysphagia Diet Standardization Initiative [IDDSI] level 0), pureed food (IDDSI level 4), and minced food (IDDSI level 5). All images were captured at the height of the swallow. The leftmost image depicts the oropharyngeal anatomy associated with the swallowing process. Part (**A**) shows silent aspiration (black arrow) and the escape of food material into the nasopharynx (white arrow). Part (**B**) shows partial improvement in soft palate elevation, epiglottis movement, tongue base retraction, and pharyngeal contraction. Minimal penetration is observed with all tested diets. Part (**C**) demonstrates complete recovery of swallowing function, returning to a normal state.

The video fluoroscopic swallowing study (VFSS) was conducted on day 6 of the illness (Figure 1A). To confirm the flow and textural characteristics of our testing material, we used the International Dysphagia Diet Standardization Initiative (IDDSI) method, which categorizes food materials into a continuum of eight levels (0–7). Her lip closure, tongue control, and mastication appeared intact during the examination. In the context of pureed foods (IDDSI level 4), a wide column of bolus was observed between the base of tongue and the posterior pharyngeal wall due to the decreased retraction of the tongue base. Minimal to no movement of the thyroid cartilage, hyoid bone, and epiglottis resulted in a wide column of contrast in the laryngeal vestibule and led to silent aspiration (PAS 8). Significant narrowing of the pharyngoesophageal segment (PES) caused minimal distention and lasted shortly, leading to marked obstruction. More than half of the food material remained within the pharyngeal structures due to decreased pharyngeal contraction. Little clearance was observed after multiple swallows. Due to decreased soft palate elevation, food material escaped to the level of nasopharynx. Silent aspiration in thin liquid without a coughing reflex was noted (IDDSI level 0, penetration/aspiration scale (PAS) 8). In the AP view, delayed vocal cord adduction was observed on the left side, and esophageal clearance appeared normal. As the symptoms progressed, follow-up VFSS was conducted on day 14. Compared to the previous examination, there were some findings that either remained similar or showed slight deterioration. Nasogastric tube feeding became necessary due to significant weight loss, with a 9 kg loss within a span of 2 weeks.

Contrast-enhanced CT of the neck ruled out structural abnormalities related to swallowing. On day 8 of the illness, laboratory studies revealed an increased white blood cell count of 12.79 × 10^9^/L, along with an elevated erythrocyte sedimentation rate (ESR) at 41 mm/h and C-reactive protein (CRP) at 2.73 mg/dL. Despite the patient reporting mild dyspnea, pulmonary function tests, maximal inspiratory and expiratory pressure, and cough peak flow were within normal limits. Based on the chest CT findings suggestive of aspiration pneumonia, antibiotics (piperacillin/tazobactam) were initiated on day 14 of the illness. On day 21 of illness, the patient began experiencing watery diarrhea, which was improved after discontinuing antibiotics. Other laboratory parameters, including thyroid function tests, tumor markers, and antibody tests for myasthenia gravis (acetylcholine receptor antibody and muscle-specific kinase antibody), yielded unremarkable results. The serological test for antiganglioside antibodies did not detect IgG or IgM antibodies against GD1b, GM1, or GQ1b. However, other antibodies, such as anti-GT1a antibody, were not available for evaluation at our hospital or nearby tertiary care centers.

Serial electrophysiological studies, including nerve conduction studies (NCSs), repetitive nerve stimulation (RNS), and needle electromyography (EMG), were conducted on days 6, 19, and 36 of the illness. These studies encompassed nerves and muscles in the face (including the bulbar region), trunk, and upper and lower limbs. On the 6th day following the onset of dysphagia, the initial study revealed no significant abnormalities, except for delayed peak latencies in the sensory nerve action potential (SNAP) of bilateral median nerves and reduced conduction velocities of the compound motor action potential (CMAP) in the right median nerve across the wrist, suggesting bilateral median entrapment neuropathy at the wrist, clinically indicative of carpal tunnel syndrome. F-wave and H-reflex results were normal in all limb muscles. RNS showed no abnormalities in proximal and distal limb muscles and facial muscles. During EMG, positive sharp waves were observed in the left mid-thoracic and bilateral upper to lower lumbar paraspinal muscles. However, no membrane instability was noted in any of the facial, tongue, and limb muscles. Quantitative analysis of EMG in all limbs were also within the normal range. Subsequent studies on days 19 and 36 revealed no evidence of progression. Results of serial electrophysiological examinations failed to diagnose any peripheral neuropathy, myopathy, or neuromuscular junction disorder that could explain the patient’s sudden dysphagia.

A cerebrospinal fluid (CSF) analysis performed on the 21st day of the illness revealed a mildly elevated total protein concentration (51 mg/dL), no white blood cells, and a normal glucose concentration (77 mg/dL), indicating albuminocytological dissociation. Following a thorough exclusion of alternative diagnoses, we consequently diagnosed the patient with an atypical variant of GBS, specifically involving cranial nerve (CN) X, and initiated intravenous immunoglobulin (IVIg) treatment for 5 days, from day 21 to day 25 of the illness. Following the initiation of IVIg treatment, the restoration of the gag reflex was observed on the third day of treatment. Significant clinical improvement was confirmed through VFSS a week after the initiation of IVIg (on day 28 from the onset), indicating a more definitive response to the therapy (Figure 1B). Soft palate elevation and tongue base retraction showed partial improvement, with a trace of contrast observed in the space between the soft palate/the base of tongue and the pharyngeal wall. Full superior movement of the thyroid cartilage and partial anterior movement of hyoid bone were observed. Additionally, with fully recovered superior movement of the thyroid cartilage and partially improved movement of the hyoid bone and epiglottis, no air or contrast was noted in the laryngeal vestibule. Minimal penetration (PAS 3) was observed across all evaluated diets, including thick pureed foods (IDDSI level 4), minced food (IDDSI level 5), soft bite-sized food (IDDSI level 6), and thin liquid (IDDSI level 0). The PES opening and pharyngeal contraction also exhibited a partial improvement, allowing a greater amount of food to pass through the esophagus with a double swallow. In the AP view, normal recovery of vocal cord adduction was observed. Consequently, the patient no longer required a nasogastric tube and began consuming pureed foods, although still experiencing difficulty with solid foods. In the VFSS conducted on day 48 of the illness, 27 days after the initiation of IVIg treatment (Figure 1C), it was confirmed that her swallowing function had fully recovered to normal, enabling her to safely consume any food without restriction.

## 3. Discussion

**Figure 2:**
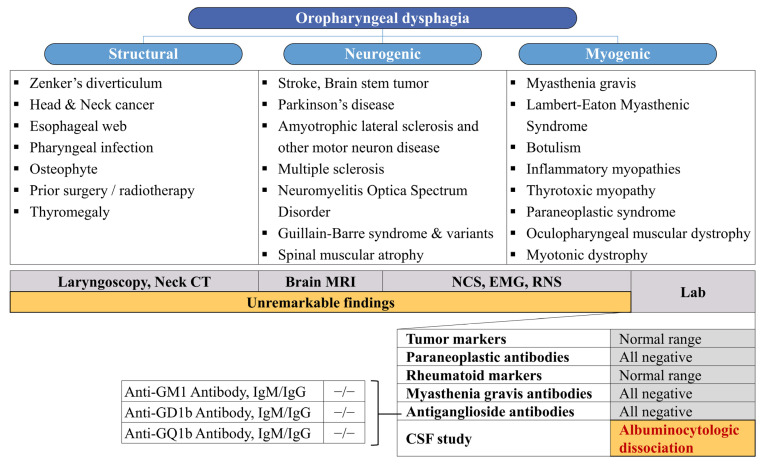
General differential diagnosis of oropharyngeal dysphagia and diagnostic approaches conducted for our patient based on this differential diagnosis.

Our patient presented with monophasic, progressive dysphagia resulting from pharyngeal muscle weakness, necessitating nasogastric tube feeding and leading to significant weight loss and aspiration pneumonia. Results from brain imaging, electrophysiologic studies (NCS, EMG, and RNS), and laboratory tests collectively indicated a low probability of other conditions, including stroke, encephalitis, peripheral polyneuropathy, myopathy, and neuromuscular junction disorders. While denervation of thoracic paraspinal muscles on EMG findings often occurs in patients with motor neuron disease, dysphagia suddenly occurred in this case, and no denervation potential was observed in the craniobulbar muscles. This ruled out motor neuron diseases such as progressive bulbar palsy or amyotrophic lateral sclerosis. Previous research has demonstrated a predominant positive reactivity for IgG anti-GT1a antibodies in ELISA evaluations of anti-ganglioside antibodies in cases of acute bulbar palsy (ABP), with IgG anti-GQ1b antibodies demonstrating a positivity rate of 55% [6]. Unfortunately, the assessment of anti-GT1a antibodies was unavailable in this case, and the anti-GQ1b antibody yielded negative results. Mild albuminocytological dissociation was observed in the CSF analysis, although the patient’s CSF total protein values, adjusted for age, did not definitively rule out the possibility of a false positive [8]. Despite some limitations, based on the clinical course, exclusion of other differential diagnoses, and response to IVIg, an atypical variant of GBS exclusively affecting the cranial nerve X was presumed as the diagnosis. Figure 2 summarizes the diagnostic approaches undertaken with our patient.

Due to the delayed diagnosis, immunotherapy was initiated on the 21st day following the onset of symptoms. However, the treatment elicited a rapid favorable response, leading to the subsequent removal of the nasogastric tube and resumption of the diet. Complete recovery of dysphagia was confirmed on the VFSS performed on day 48 of onset. These observations suggest that the patient’s bulbar palsy was likely caused by an immune-mediated mechanism. In this case, the reasons for the delay in diagnosis and treatment included (1) consideration of more highly prevalent diseases, such as stroke, due to her medical history and old age; (2) absence of antecedent illnesses such as diarrhea, respiratory infection, or vaccination; (3) normal neurological examination except for dysphagia and decreased gag reflex; and (4) normal nerve conduction findings in serial examinations. Despite not being included within the diagnostic criteria of GBS, up to 76% of patients present with an antecedent illness in the preceding four weeks [5]. Although our patient was treated for aspiration pneumonia, no evidence of infection was found in the laboratory and radiologic findings performed at the emergency room at the time of dysphagia onset, suggesting that aspiration pneumonia developed as dysphagia progressed.

ABP can manifest in various subtypes within the disease spectrum of GBS; however, most cases are accompanied by other neurological abnormalities, such as ascending limb weakness (classic GBS) or cervical-brachial weakness (PCB variant) [5,9]. The case reports previously published regarding acute dysphagia diagnosed as a variant of GBS are summarized in Table 1.

In a previous nationwide survey, the Korean Inflammatory Neuropathy Consortium reported 11 cases with prominent ABP without limb weakness among 184 GBS cases and proposed the ABP-plus syndrome as a variant of GBS [6]. In this report, the mean age at onset was 33.8 years (range 18–65 years), which was notably younger than our case. The patients in the report exhibited additional GBS features including ophthalmoplegia, sensory ataxia, or minor abnormalities in NCS. However, in our patient, there were no neurological signs or symptoms other than dysphagia, making it a highly unusual case. The clinical symptom localized to the pharyngeal muscles in this case may be explained by the patient’s relatively low CSF total protein level of 51 mg/dL, given the diagnostic threshold of the albuminocytologic dissociation (>45 mg/dL). A previous study has reported that low levels of CSF total protein are observed in patients with normal NCS or the Miller Fisher syndrome, rather than in cases of classic GBS [15].

Elderly patients presenting with dysphagia demonstrate a range of etiologies and clinical courses, either as an isolated symptom or alongside other manifestations. Dysphagia in the elderly constitutes a critical condition that can substantially increase patient morbidity and mortality, even over a short duration [16]. With a thorough evaluation of systems impacting the swallowing mechanism and awareness of uncommon causative factors, such as neuro-autoimmunity, clinicians can implement effective disease-modifying therapies, potentially leading to the resolution of dysphagic symptoms.

## 4. Conclusions

This case report presents a rare variant of GBS, in which the patient achieved complete recovery through immunotherapy. It highlights the challenges in diagnosing and treating atypical types of GBS in elderly individuals. Therefore, it is crucial for investigators to conduct a comprehensive evaluation of its potential causes to determine appropriate therapeutic interventions.

## Figures and Tables

**Table 1:** Clinical characteristics of patients in previous case reports of acute dysphagia diagnosed as a variant of GBS.

Case	Age/Sex	Antecedent Infection	Clinical Manifestations Other than Dysphagia	Nasogastric Tube Insertion	EDX	Antiganglioside Antibodies	Albuminocytoloic Dissociation	Treatment	Outcome
Onodera et al. [10]	29/M	Diarrhea	Facial palsy, neck weakness, dysarthria	UC	NT	IgG anti-GT1a, GQ1b	+	Plasmapheresis	FR
Hamidon et al. [9]	19/F	None	Areflexia	+	Abn (DML)	NT	+	None	FR
Mehta et al. [11]	48/M	None	Areflexia	+	Abn (DML, CV, CMAP, SNAP)	IgM anti-GM2	+	None	FR
Hwang et al. [12]	63/M	None	Facial palsy, external OP, dysarthria, areflexia, truncal ataxia	UC	Normal	IgG anti-GT1a, GQ1b	−	IVIg	Mild OP
Cao et al. [13]	52/F	URI	External OP, hyporeflexia	+	Abn (SNAP)	IgG anti-GQ1b, GT1a	+	IVIg	Mild blurred vision
Mansour et al. [14]	56/M	URI	Aphonia, tetraplegia, areflexia, sensory ataxia	UC	Abn (DML, CV, sensory CV)	NT	+	IVIg, MPD, Plasmapheresis	FR

Abbreviations: EDX = electrodiagnostic study; UC = uncheckable; NT = not tested; IgG = immunoglobulin G; FR = full recovery; Abn = abnormal; DML = distal motor latency; CV = conduction velocity; CMAP = compound motor action potential; SNAP = sensory nerve action potential; IgM = immunoglobulin M; OP = ophthalmoplegia; IVIg = intravenous immunoglobulin; URI = upper respiratory tract infection; MPD = Methylprednisolone.

## Data Availability

The data presented in this study are available upon request from the corresponding author.
